# An Effective Hypoxia-Related Long Non-Coding RNAs Assessment Model for Prognosis of Clear Cell Renal Carcinoma

**DOI:** 10.3389/fonc.2021.616722

**Published:** 2021-02-22

**Authors:** Han Zhang, Chuan Qin, Hua Wen Liu, Xiong Guo, Hua Gan

**Affiliations:** ^1^ Department of Nephrology, The First Affiliated Hospital of Chongqing Medical University, Chongqing, China; ^2^ Department of Oncology, Chongqing University Three Gorges Hospital, Chongqing, China; ^3^ Department of Gastrointestinal Surgery, Chongqing University Three Gorges Hospital, Chongqing, China; ^4^ Department of Gastrointestinal Surgery, The First Affiliated Hospital of Chongqing Medical University, Chongqing, China

**Keywords:** clear cell renal carcinoma, hypoxia, long non-coding RNA, prognosis, biomarker

## Abstract

Hypoxia is a significant clinical feature and regulates various tumor processes in clear cell renal carcinoma (ccRCC). Increasing evidence has demonstrated that long non-coding RNAs (lncRNAs) are closely associated with the survival outcomes of ccRCC patients and regulates hypoxia-induced tumor processes. Thus, this study aimed to develop a hypoxia-related lncRNA (HRL) prognostic model for predicting the survival outcomes in ccRCC. LncRNAs in ccRCC samples were extracted from The Cancer Genome Atlas database. Hypoxia-related genes were downloaded from the Molecular Signatures Database. A co-expression analysis between differentially expressed lncRNAs and hypoxia-related genes in ccRCC samples was performed to identify HRLs. Univariate and multivariate Cox regression analyses were performed to select nine optimal lncRNAs for developing the HRL model. The prognostic model showed good performance in predicting prognosis among patients with ccRCC, and the validation sets reached consistent results. The model was also found to be related to the clinicopathologic parameters of tumor grade and tumor stage and to tumor immune infiltration. In conclusion, our findings indicate that the hypoxia-lncRNA assessment model may be useful for prognostication in ccRCC cases. Furthermore, the nine HRLs included in the model might be useful targets for investigating the tumorigenesis of ccRCC and designing individualized treatment strategies.

## Introduction

Renal cell carcinoma (RCC) causes more than 100,000 deaths per year (1). Although target therapy and immunotherapy have improved the prognosis of RCC patients (2), the 5-year survival rate remains less than 10%. Clear cell renal cell carcinoma (ccRCC) is the main subtype of RCC, accounting for 70–75% of all RCC cases (3). In clinical practice, the prognosis and treatment of ccRCC are primarily based on the tumor stage. However, the outcomes still vary among patients with the same tumor stage because of molecular heterogeneity (4). Therefore, it is vital to identify individualized biomarkers that can identify patients at high risk of death and help stratify patients for individual treatment to optimize the therapeutic effect.

Hypoxia refers to a reduction of oxygen availability at the cell level, including in tumors (5). As a significant clinical feature, hypoxia regulates various tumor processes, including angiogenesis, cell proliferation, invasion, apoptosis, and radiochemotherapy resistance (6). Hypoxia adaption is a key factor in tumor progression and has been proven to be a cause of treatment failure (7).

Long non-coding RNAs (lncRNAs) are untranslated RNAs of >200 nucleotides in length (8). They have recently attracted increasing research attention because of their involvement in several key molecular and biologic processes (9, 10). For example, lncRNAs regulate hypoxia-related tumor processes (11). In RCC, lncRNA-SARCC can regulate tumor cell proliferation through the androgen receptor/HIF-2*α*/C-MYC axis under hypoxia (12). lncRNA EGOT can also regulate autophagy under hypoxia in renal tubular cells (13). Therefore, a hypoxia-related lncRNA (HRL)-based prognostic model may be potentially useful in ccRCC.

As such, this study aimed to develop a HRL prognostic model for predicting the survival outcomes in ccRCC.

## Materials and Methods

### Data Source

Transcriptome expression profiles for patients with ccRCC were obtained from The Cancer Genome Atlas database ((TCGA), https://cancergenome.nih.gov/) on June 29, 2020 (14). The expressions were quantified with fragments per kilobase of exon per million reads mapped. The corresponding clinical information of the patients from whom the samples were obtained was also downloaded from the database, which included age, sex, tumor grade, tumor stage, and survival (**Table 1**). Patients with incomplete information or <30 days of data were excluded because they might have died because of acute complications, rather than of the cancer itself.

**Table 1:** Baseline patient characteristics (n = 537).

Characteristic	537 clear cell renal carcinoma patients
**Age**	
<=65 years	352(66%)
>65 years	185(34%)
Unknown	0(0%)
**Sex**	
Female	191(36%)
Male	346(64%)
Unknown	0(0%)
**Tumor Grade**	
1&2	244(45%)
3&4	285(53%)
Unknown	8((2%))
**Tumor Stage**	
I	269(50%)
II	57(10.5%)
III	125(23%)
IV	83(15.5%)
Unknown	3(0.5%)
**Pathologic T Stage**	
T1&2	344(64%)
T3&4	193(36%)
Unknown	0(0%)
**Pathologic N Stage**	
N0	240(45%)
N1	17(3%)
Unknown	280(52%)
**Pathologic M Stage**	
M0	426(79%)
M1	79(15%)
Unknown	32(6%)

Data on hypoxia-related genes were collected from the Molecular Signatures Database V7.2 (https://www.gsea-msigdb.org/gsea/msigdb, Hypoxia M10508, Hypoxia cancer M7547) (15). If the expression data of the gene are not detected in more than 50% of the samples, the gene is excluded. Immune infiltration data were collected from CIBERSORT (https://cibersort.stanford.edu/) (16), which contains abundances of 22 types of tumor-infiltrating immune cells, namely, naive B cells, memory B cells, plasma cells, CD8 T cells, naive CD4 T cells, resting memory CD4 T cells, activated memory CD4 T cells, follicular helper T cells, T cells regulatory, gamma delta T cells, resting NK cells, activated NK cells, monocytes, macrophages M0, macrophages M1, macrophages M2, resting dendritic cells, activated dendritic cells, resting mast cells, activated mast cells, eosinophils, and neutrophils.

### Definition of Hypoxia-Related Long Non-Coding RNAs

Genes were identified as protein-coding genes or lncRNAs according to their Ensembl IDs. The lncRNAs were further screened *via* the Genecards database (https://www.genecards.org/) (17). We excluded the lncRNAs recognized as “Pseudogene,” “Uncategotized,” and “No results” in the database. Differentially expressed lncRNAs between the kidney and healthy renal tissue were identified *via* the differential-expression analysis using the R package “limma” (log2 fold-change [logFC] of >1 and an adjusted false-discovery rate [FDR] of <0.05) (18). Heatmaps and volcano plots were used to visualize the differentially expressed lncRNAs *via* the R package “pheatmap.” (19)

We then performed co-expression analysis between hypoxia genes and differentially expressed lncRNAs based on the Spearman correlation analysis (20, 21). LncRNAs with a Spearman correlation coefficient ≥0.4 and a P-value ≤0.001 were identified as HRLs.

### Development of the Hypoxia Long Non-Coding RNA-Related Prognostic Model

All the samples were randomly divided into the training dataset and the 1^st^ validation dataset at the ratio of 1:1. Then the samples were randomly divided into the 2^nd^ validation dataset and 3^rd^ validation dataset at the ratio of 3:7. The training dataset was used to construct the HRL-related prognostic model to predict the prognosis for ccRCC patients. Univariate Cox regression analyses were used to extract the hypoxia survival-associated lncRNAs *via* the R package “survival” (significant at P ≤ 0.01). A Cox proportional hazards model with a lasso penalty analysis was used to construct the HRL model with the optimal prognostic value *via* the R packages “glmnet” and “survival.” (22) The risk score of each sample was calculated based on the regression coefficients from the model and lncRNAs’ expression. The formula is below:

$$\mathrm{Risk}\text{ score (patient)=}\sum_{k=1}^{n}\left(coef\times exp\right)$$

with “n” representing the number of lncRNA; “k,” the serial number of each lncRNA; coef, the coefficient value from the Cox proportional hazards analysis; and exp, the expression of the lncRNA (23).

### Validation of the Model

The validation datasets were used to validate the predictive power of the HRL-related model. In each dataset, patients were assigned to the low- and high-risk groups based on the median risk scores. Kaplan–Meier survival curve analyses and log-rank tests were performed to evaluate the predictive power of the model for overall survival (OS), using the R package “survival” and “survminer.” Receiver operating characteristic (ROC) curves (24) and area under the ROC curves (AUC) were calculated to assess the accuracy of the model, using the R package “survivalROC.” An AUC of >0.75 was judged as excellent predictive value. Univariate and multivariate analyses *via* the R package “survival” were also performed to verify the independent prognostic predictors. The nomogram was plotted using the R package “rms.” (25)

### Gene Set Enrichment Analysis

Gene set enrichment analysis (GSEA) (version 4.0.1, http://www.broadinstitute.org/gsea) was performed to identify differences in the set of genes expressed between the low- and high-risk groups in the enrichment of Kyoto Encyclopedia of Genes and Genomes (KEGG) and Gene Ontology (GO) data. Gene set permutations were performed 1,000 times for each analysis.

### Statistical Analysis

All statistical analyses were performed using the R software (version 3.6.1, http://www.R-project.org). The PERL programming language (version, 5.30.2, http://www.perl.org) was used to process data. The Wilcoxon signed-rank test was used for identifying differentially expressed lncRNAs and tumor-infiltrating immune cells. The Spearman correlation analysis was used for identifying HRLs. The Kaplan–Meier method and log-rank test were performed to compare the OS between the high- and low-risk groups.

## Results

### Hypoxia-Related Long Non-Coding RNAs in Clear Cell Renal Carcinoma

**Figure 1:**
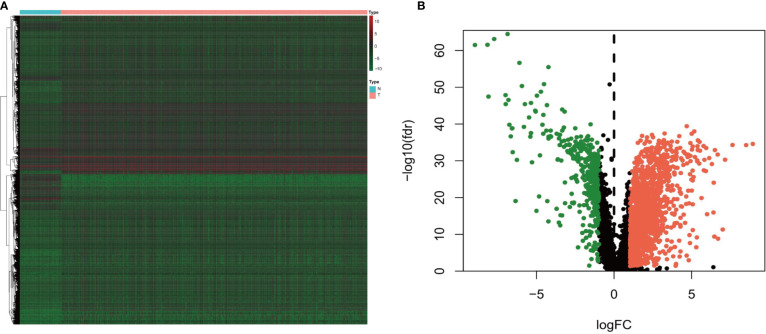
**(A)** Heatmap and **(B)** volcano diagram of the Wilcoxon signed-rank test showing the differentially expressed lncRNAs between clear cell renal carcinoma and normal tissue samples. The red, green, and black dots represent the upregulated lncRNAs, downregulated lncRNAs, and no difference, respectively.

A total of 14,143 lncRNAs were extracted from the TCGA database. We identified 1,926 differentially expressed lncRNAs in renal cancer specimens (n = 539) and normal renal specimen (n = 72) (logFC of >1 and FDR of <0.05) (**Figures 1A, B**). Among these lncRNAs, 186 lncRNAs were excluded due to the lack of definition in the Genecards database.

Of the 137 hypoxia genes obtained from the Molecular Signatures Database V7.2, four genes (FGF3, LIN28B, MMP13, and TH) were excluded owing to a lack of over 50% expression information. In total, 598 HRLs were confirmed by co-expression analyses between hypoxia genes and differentially expressed lncRNAs (P ≤ 0.001, Spearman correlation coefficient ≥0.4).

### Construction of Hypoxia Long Non-Coding RNA-Related Prognostic Model

After excluding patients without cancer or survival data, we merged the survival data with lncRNA expression data of each patient. We then divided the remaining patients into the training dataset (n = 255) and the 1^st^ validation dataset (n = 252) at the ratio of 1:1 and divided the patients into the 2^nd^ validation dataset (n = 153) and the 3^rd^ validation dataset (n = 354) at the ratio of 3:7. The risk model was developed using the training dataset and validated using the validation datasets.

**Figure 2:**
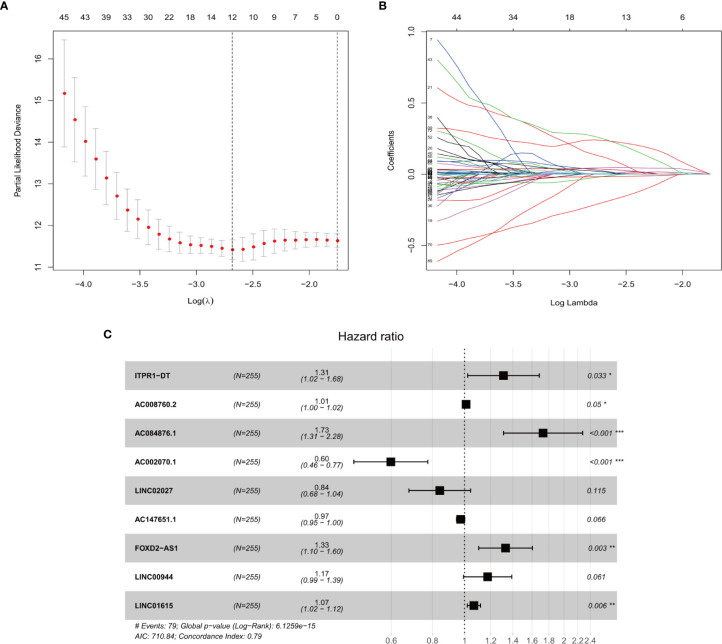
**(A, B)** The LASSO Cox regression model to identify the most robust lncRNAs. **(C)** Forest plot of the multivariate Cox regression model showing the nine optimal prognostic hypoxia-related lncRNAs. * represents p < 0.05, ** represents p < 0.01, *** represents p < 0.001.

Univariate cox regression analyses were first performed for the hypoxia differentially expressed lncRNAs, and the results showed that 163 lncRNAs were significantly related to the OS of ccRCC (P ≤ 0.01). A Cox proportional hazards model with a lasso penalty analysis was further performed to construct the optimal risk model (**Figures 2A, B**). Ultimately, nine optimal prognostic HRLs were obtained and incorporated into the risk model: ITPR1-DT, AC008760.2, AC084876.1, AC002070.1, LINC02027, AC147651.1, FOXD2-AS1, LINC00944, and LINC01615 (**Figure 2C**). The risk score for each patient was calculated as: risk score = (0.271 × ITPR1-DT expression) + (0.011 × AC008760.2 expression) + (0.546 ×AC084876.1expression) + (−0.514 × AC002070.1 expression) + (−0.173 × LINC02027 expression) + (−0.027 × AC147651.1 expression) + (0.286 × FOXD2-AS1 expression) + (0.161 × LINC00944 expression) + (0.065 × LINC01615 expression).

### Validation of the Prognostic Score

**Figure 3:**
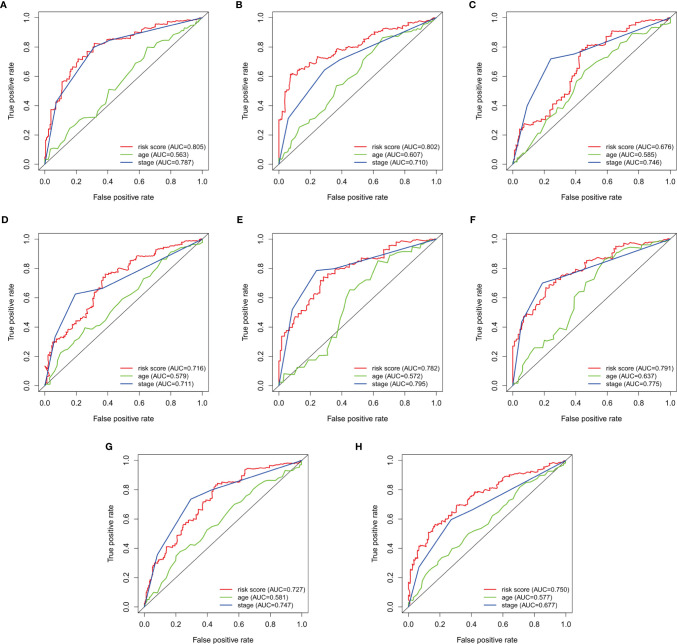
Survival-dependent receiver operating characteristics (ROC) for predicting survival in the datasets. ROC for predicting the **(A)** 3-year, and **(B)** 5-year survival in the training dataset. ROC for the **(C)** 3-year, and **(D)** 5-year survival in the 1^st^ validation dataset. ROC for predicting the **(E)** 3-year, and **(F)** 5-year survival in the 2^nd^ dataset. ROC for predicting the **(G)** 3-year, and **(H)** 5-year survival in the 3^rd^ validation dataset.

To verify the accuracy of prognostic prediction of each patient, we performed ROC in the training dataset and the validation datasets. In the training dataset, the AUCs for predicting the 3-, and 5-year survival were 0.805, and 0.802, respectively, indicating excellent prognostic power (**Figures 3A, B**). Similar results were obtained in the 1^st^ (**Figures 3C, D**), 2^nd^ (**Figures 3E, F**) and 3^rd^ (**Figures 3G, H**) validation datasets.

**Figure 4:**
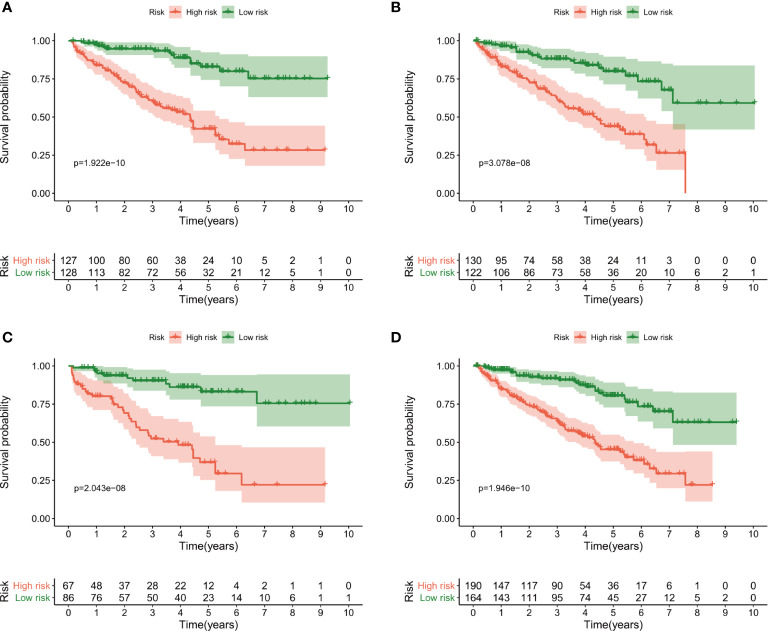
Kaplan–Meier curves of overall survival for the high-risk and low-risk groups according to the median risk score. **(A)** The training dataset, **(B)** 1^st^ validation dataset, **(C)** 2^nd^ validation dataset and **(D)** 3^rd^ validation dataset.

The patients were then divided into the high- and low-risk groups using the median risk score as a cut-off. Kaplan–Meier curves were plotted in the training dataset, and the results showed poorer survival in the high-risk group than in the low-risk group (P = 1.922e-10) (**Figure 4A**). The survival analyses in the validation groups also revealed poorer survival in the high-risk groups than in the low-risk groups [3.078e-08 in the 1^st^ (**Figure 4B**), 2.043 e-08 in the 2^nd^ (**Figure 4C**), and 1.946e-10 in the 3^rd^ validation dataset (**Figure 4D**)].

**Figure 5:**
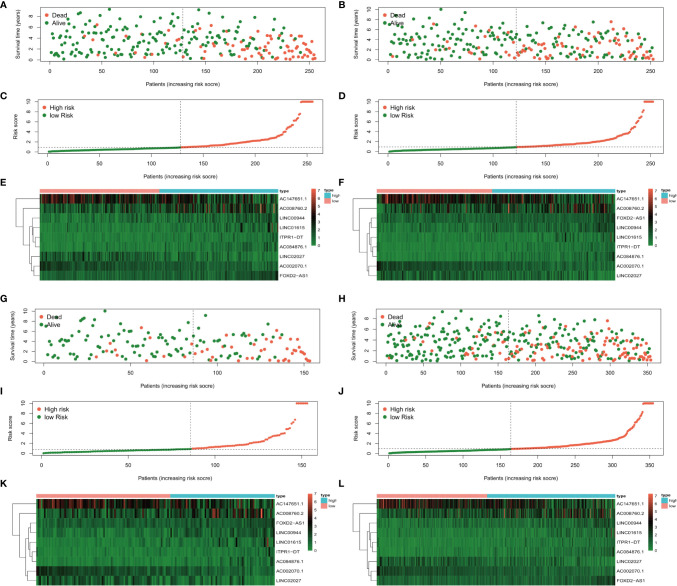
The survival status, risk score distribution, and risk lncRNA expression in the datasets. **(A, C, E)** Training dataset, **(B, D, F)** 1^st^ validation dataset, **(G, I, K)** 2^nd^ validation dataset, and 3^rd^ validation dataset **(H, J, L)**.

The risk score distributions, survival status, and risk gene expressions in each dataset are shown in **Figure 5**. The low-risk groups had obviously higher survival rate (**Figure 5A**) and lower values for the risk score (**Figure 5C**) in the training dataset. Moreover, as the risk score increased, the expressions of the protective lncRNAs (AC008760.2, LINC00944, LINC01615, ITPR1-DT, AC084876.1, and FOXD2-AS1) decreased, whereas those of the risk lncRNAs (AC147651.1, LINC02027, and AC002070.1) increased (**Figure 5E**) in the training dataset. Similar results were obtained in the 1^st^ (**Figures 5B, D, F**), 2^nd^ (**Figures 5G, I, K**) and 3^rd^ (**Figures 5H, J, L**) validation datasets.

**Figure 6:**
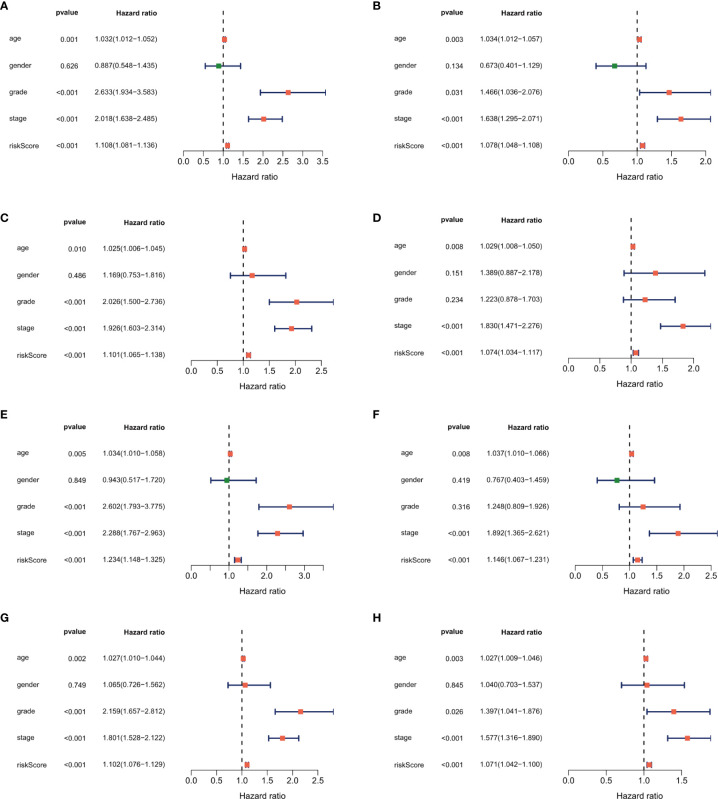
Forest plot of the univariate and multivariate Cox regression analysis showing that the risk score was an independent risk factor for overall survival in the training dataset **(A, B)**, 1^st^ validation dataset **(C, D)**, 2^nd^ dataset **(E, F)** and the 3^rd^ validation dataset **(G, H)**.

In the univariate analysis to evaluate the relationship between clinical characteristics and OS, the TNM stage was excluded because several patients had missing information. The results showed that age (P = 0.003), tumor grade (P = 0.031), tumor stage (P < 0.001), and risk score (P < 0.001) were significantly associated with prognosis (**Figures 6A, C, E, G**). Multivariate analysis confirmed age, tumor stage, and risk score as independent prognostic factors (**Figures 6B, D, F, H**). In addition to risk score, age and tumor stage could also divide patients into high- and low-risk groups effectively (**Supplementary Figure 1**). To further verify the predictive power of our risk score in the patients with same tumor stage, we divided early stage (I and II) and advanced stage (III and IV) ccRCC patients into the high- and low-risk groups using the median risk score. Kaplan–Meier curves were plotted in two groups, and the results showed poorer survival in the high-risk groups than in the low-risk groups (**Supplementary Figure 2**).

**Figure 7:**
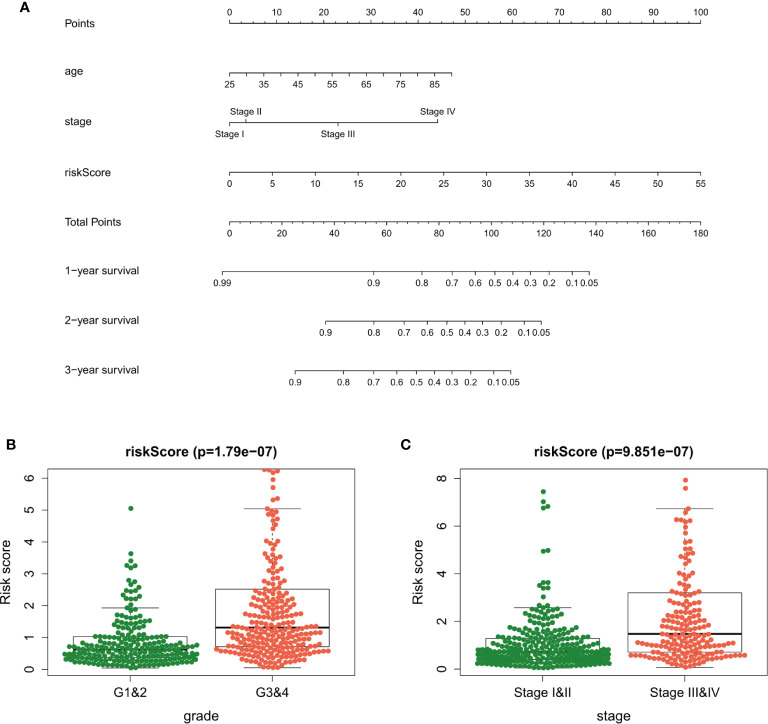
A nomogram plot was established to qualify risk assessment for ccRCC patients **(A)**. Relationships between the risk score and clinical factors [tumor grade **(B)** and tumor stage **(C)**] in clear cell renal carcinoma.

The independent prognostic factors (age, tumor stage, and risk score) were used to develop the nomogram for predicting the 1-, 3-, and 5-year prognoses of the patients (**Figure 7A**). Similar results were obtained in the validation datasets (**Supplementary Figure 3**). In the nomogram, we can calculate the point of each factor and the total points of all factors. The 1-, 3-, and 5-year survival rates could be predicted by the corresponding value of total points.

### Clinical Utility of the Risk Score

The association among the risk lncRNAs (ITPR1-DT, AC008760.2, AC084876.1, AC002070.1, LINC02027, AC147651.1, FOXD2-AS1, LINC00944, and LINC01615), risk score, and clinicopathologic parameters (age, sex, tumor grade, and tumor stage) was analyzed in the training dataset (**Table 2**). The risk score was obviously higher in samples with high-grade and advanced-stage tumor (**Figures 7B, C**). Similar results were obtained in the validation datasets (**Supplementary Tables**). This finding supports that the risk score can also reflect tumor progression.

**Table 2:** Relationships of the risk score and the risk genes with clinical variables in ccRCC.

lncRNA symbol	Age(≤65/>65)	Sex(male/female)	Tumor grade(1&2/3&4)	Tumor stage(I & II/III & IV)
ITPR1-DT	0.89(0.374)	−0.152(0.879)	−1.907(0.058)	−1.454(0.148)
AC008760.2	1.779(0.077)	1.179(0.241)	−2.858(0.005)	−1.62(0.108)
AC084876.1	−1.782(0.077)	0.062(0.951)	−2.83(0.005)	−3.251(0.001)
AC002070.1	0.86(0.391)	0.86(0.391)	5.216(4.132e-07)	3.722(2.446e-04)
LINC02027	−0.991(0.323)	2.373(0.019)	1.248(0.213)	1.076(0.283)
AC147651.1	0.522(0.602)	1.313(0.191)	4.319(2.462e-05)	2.032(0.043)
FOXD2-AS1	−0.575(0.567)	0.473(0.637)	−2.183(0.030)	−3.833(1.836e-04)
LINC00944	−1.54(0.125)	−1.947(0.053)	−3.653(3.219e-04)	−4.291(2.922e-05)
LINC01615	0.994(0.321)	−1.957(0.052)	−2.12(0.036)	−2.276(0.025)
Risk Score	−0.563(0.574)	0.438(0.662)	−4.646(7.756e-06)	−3.993(1.171e-04)

lncRNA, long non-coding RNA.

**Figure 8:**
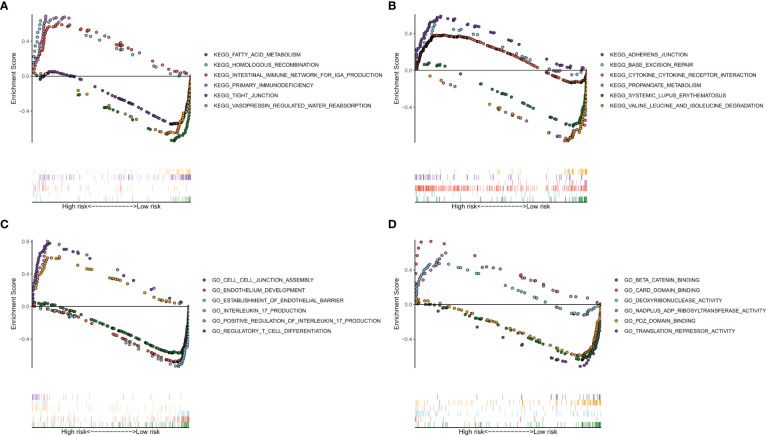
Analysis of enriched pathways. KEGG analysis **(A, B)** of Gene Set Enrichment Analysis in the high- and low-risk groups in clear cell renal carcinoma. GO analysis **(C, D)** of Gene Set Enrichment Analysis in high- and low-risk groups in clear cell renal carcinoma.

To explore which pathways were enriched, we used GSEA software to perform KEGG (**Figures 8A, B**) and GO analysis (**Figures 8C, D**). KEGG analysis identified multiple tumor-related signaling pathways in the high-risk group, such as homologous recombination, Base excision repair, and cytokine-cytokine receptor interaction. Surprisingly, KRGG and GO analysis identified that several immune-related signal pathways and genes were enriched in the samples.

**Figure 9:**
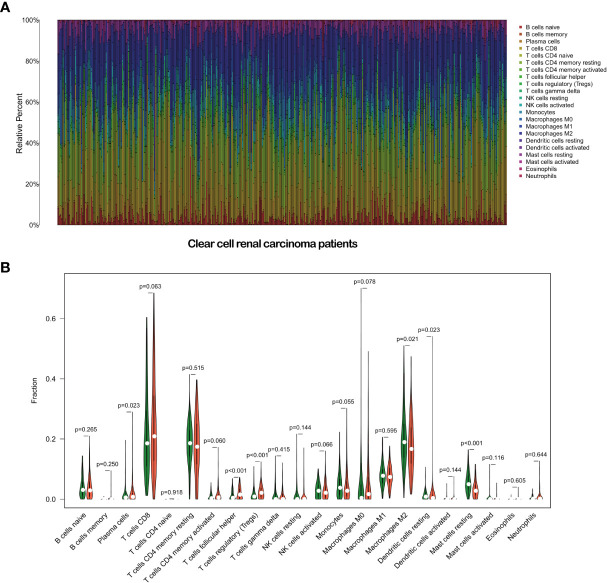
Immune landscape of the patients with clear cell renal carcinoma **(A)**. Relationships between the risk score and the immune cell infiltration in clear cell renal carcinoma **(B)**.

We further analyzed the correlation between immune cell infiltration and the risk score. First, we plotted the immune landscape of all the samples, as shown in **Figure 9A**. Then, we analyzed the difference in the number of immune cells between the low- and high-risk groups for all the samples. We identified six types of immune cells with differences in infiltration between the two groups, namely, plasma cells, follicular helper T cells, regulatory T cells, M2 macrophages, resting dendritic cells, and resting mast cells (**Figure 9B**).

## Discussion

Despite advances in diagnosis and treatment, ccRCC as a lethal RCC subtype remains to have poor prognosis (26). Further, current prognostic models for ccRCC have limited predictive capability because of the complex molecular heterogeneity of this malignancy. Hence, in this study, we identified a novel prognostic model for predicting ccRCC outcomes.

Hypoxia has been confirmed to be closely related to tumorigenesis and tumor progression of ccRCC (27). Previous studies have established that lncRNAs are involved in tumorigenesis, tumor progression, and metastasis (28–30). In this study, HRLs were related to the survival outcomes of patients with ccRCC, and thus we developed an HRL-related model to predict ccRCC prognosis. To our best knowledge, this is the first study to develop such predictive model for ccRCC.

Previous studies suggested that lncRNAs are involved in multiple processes in ccRCC (31, 32). For example, lncRNA UCA1 plays an oncogenic role in RCC by regulating the miR-182-5p/DLL4 axis (33). LncRNA URRCC can also promote the proliferation and metastasis of ccRCC by regulating the P-AKT signaling pathway (34). Further, lncRNA OTUD6B can inhibit ccRCC cell proliferation by suppressing the Wnt/*β*-catenin pathway and the expressions of epithelial-to-mesenchymal transition-related proteins (35). In this study, we screened 1926 differentially expressed lncRNAs in ccRCC tissue, relative to the levels in adjacent normal renal tissue. The results indicated that lncRNAs are closely related to the tumorigenesis of ccRCC, in agreement with previous findings.

Tumor hypoxia is defined as lower oxygenation in solid tumors than in normal tissues. Hypoxia can lead to resistance to chemoradiotherapy and target therapy (7, 36); increases angiogenesis and vasculogenesis (37), thus predisposing to tumor metastases; and contributes to altered metabolism and genomic instability. LncRNAs have been reported be involved in the development of ccRCC by regulating the hypoxia pathway. For example, Hamilton et al. found that lncRNA HOTAIRM1 inhibited the hypoxia pathway in ccRCC (38). Zhang et al. revealed that under hypoxic conditions in ccRCC, lncRNA CRPAT4 promoted cell migration by regulating AVL9 (39). In the present study, we performed a co-expression analysis between hypoxia genes and differentially expressed lncRNAs through paired lncRNA and mRNA expression data in ccRCC patients from TCGA. A total of 598 lncRNAs were extracted and defined as HRLs. The close association between hypoxia genes and HRLs in ccRCC samples indicate that HRLs are involved in the development of ccRCC.

Among all the HRLs, nine lncRNAs (*i.e*., ITPR1-DT, AC008760.2, AC084876.1, AC002070.1, LINC02027, AC147651.1, FOXD2-AS1, LINC00944, and LINC01615) were identified to be independently associated with prognosis and were thus used to develop the prognostic model. ROC curves confirmed the good specificity and sensitivity of the HRL-based prognostic model. Kaplan-Meier survival curves showed excellent efficiency of our HRL-related model in stratifying patients with different risks of mortality. Multivariate analyses demonstrated that the age, tumor stage and the risk score were independent prognostic factors. We further identified the prognostic predictive power of our risk score in the patients with same tumor stage. Hence, our HRL-related model maybe useful as a supplement to the tumor stage for better stratifying patients and for providing a more individualized approach to treatment. We further developed a nomogram by integrating age, tumor stage, and risk score. From it we can easily obtain a single number, which reflects survival when accounting for these three factors.

Tumor hypoxia also changes the interaction and cross-talk of cancer cells with the surrounding tumor microenvironment, leading to immune resistance and immune suppression, which help tumor cells escape immune surveillance (5, 40, 41). To determine whether our HRL-related model can also reflect the tumor microenvironment, we performed GSEA. The results showed that several immune-related GO terms or signaling pathways were enriched in the high-risk group. We further plotted the immune landscape of each ccRCC sample for exploring the tumor immune microenvironment in patients with ccRCC. Then, we compared the infiltration of every immune cell type between the high- and low-risk groups. Plasma cells, follicular helper T cells, regulatory T cells, M2 macrophages, resting dendritic cells, and resting mast cells were found to be differentially infiltrated in ccRCC, which are closely associated with tumorigenesis, progression, and metastasis (42–46). This finding supports that our HRL-related model can partly reflect immune infiltration and provide valuable information for immunotherapy.

The whole process of our analyses was based on the data from TCGA database, which contains complete clinical and survival data of patients with ccRCC. It also has sufficient ccRCC samples to be divided into a training dataset and validation datasets. Therefore, a prognostic model constructed using TCGA database has better statistical power than a model constructed using patient samples derived from a single institution. However, the current study still has some limitations. First, we haven’t found an available independent lncRNA dataset to validate the usefulness of our prognostic model, and we were not able to validate in clinical practice owing to the lack of ccRCC samples. Second, the relationship between the nine lncRNAs and ccRCC remains unclear to date because of the limited number of lncRNA researches. The validity of our prognostic model should be evaluated in further research with a large number of clinical samples and with adequate follow-up duration. In addition, the underlying mechanisms by which lncRNAs influence the prognosis of ccRCC should be investigated in *in vivo* and *in vitro* experiments.

## Conclusion

Our hypoxia-lncRNA assessment model may be useful to improve the prognostic prediction of ccRCC patients with the same tumor stage. Furthermore, the nine HRLs included in the model might be useful targets for investigating the tumorigenesis of ccRCC and designing personalized individualized treatment strategies.

## Data Availability Statement

Publicly available datasets were analyzed in this study. These data can be found here: The Cancer Genome Atlas (https://portal.gdc.cancer.gov/), the Molecular Signatures Database V7.2 (https://immport.niaid.nih.gov), CIBERSORT (https://cibersort.stanford.edu/).

## Author Contributions

HZ and HG designed the study. HZ and CQ collected and analyzed the data, and drafted the manuscript. CQ and HL made the figures and tables. XG provided critical suggestions regarding the figures and manuscript. HG led the research team. All authors contributed to the article and approved the submitted version.

## Conflict of Interest

The authors declare that the research was conducted in the absence of any commercial or financial relationships that could be construed as a potential conflict of interest.

## Supplementary Material

Supplementary Figure 1
**(A)** Kaplan–Meier curves of overall survival for the young (≤50 y) and elderly (>50 y) groups. **(B)** Kaplan–Meier curves of overall survival for the early (I–II) and advanced (III–IV) tumor stage groups.

Supplementary Figure 2 Kaplan–Meier curves of overall survival for the highrisk and low-risk groups according to the median risk score. **(A)** ccRCC patients with early tumor stages (I–II) and **(B)** advanced stages (III–IV).
