# Supplementary figures and images for: An Effective Hypoxia-Related Long Non-Coding RNAs Assessment Model for Prognosis of Clear Cell Renal Carcinoma
Source: Front Oncol. 2021 Feb 22;11:616722. doi: 10.3389/fonc.2021.616722 (PMC7937891; doi:10.3389/fonc.2021.616722)

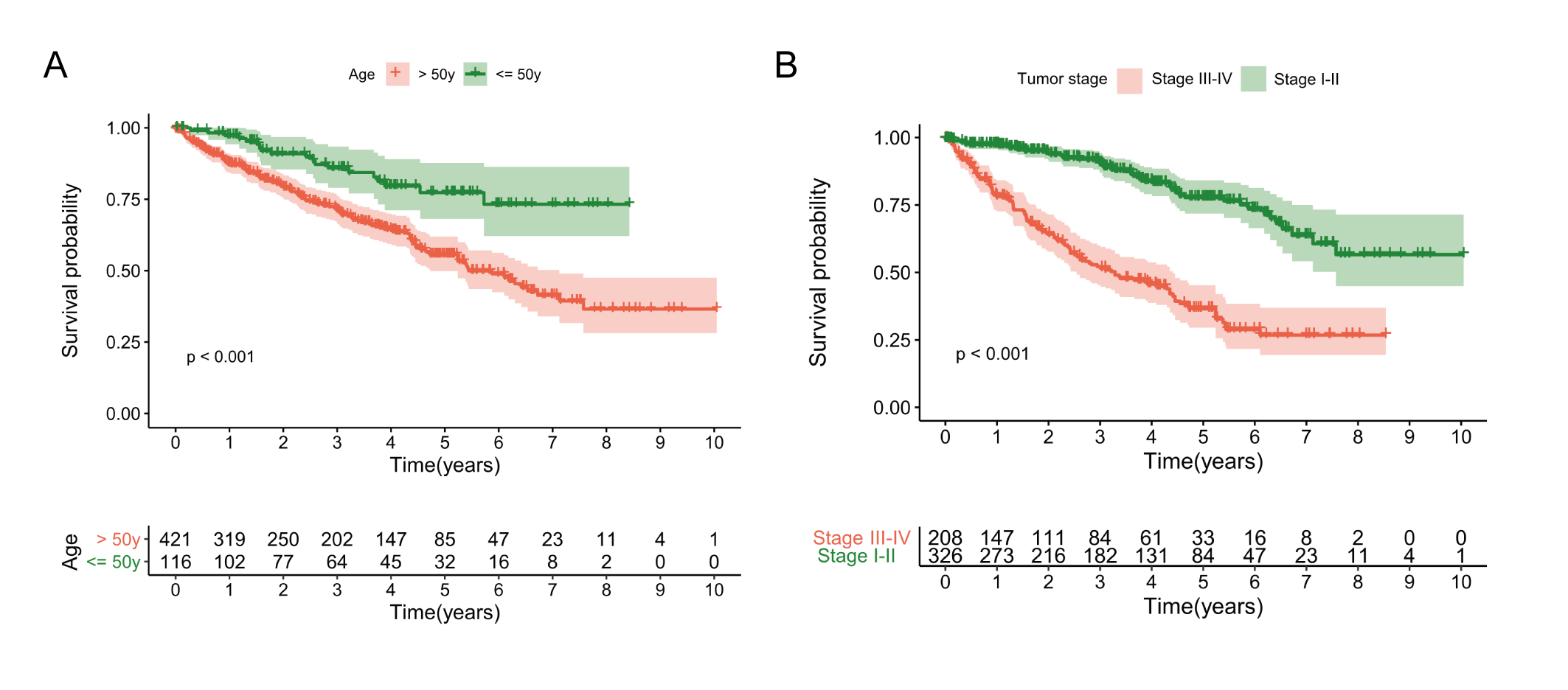

Supplement: Supplementary Figure 1 — (A) Kaplan–Meier curves of overall survival for the young (≤50 y) and elderly (>50 y) groups. (B) Kaplan–Meier curves of overall survival for the early (I–II) and advanced (III–IV) tumor stage groups. [file Image_1.tif]

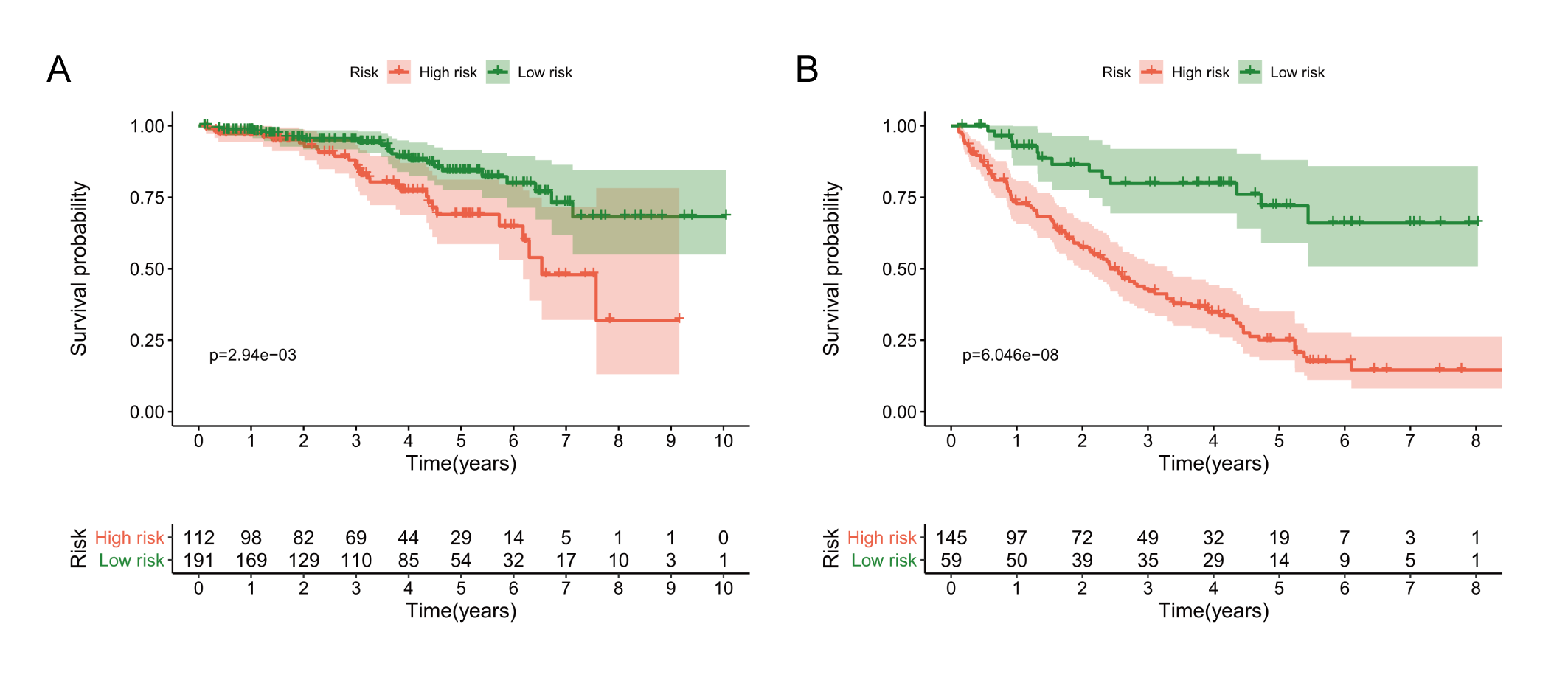

Supplement: Supplementary Figure 2 — Kaplan–Meier curves of overall survival for the highrisk and low-risk groups according to the median risk score. (A) ccRCC patients with early tumor stages (I–II) and (B) advanced stages (III–IV). [file Image_2.tif]

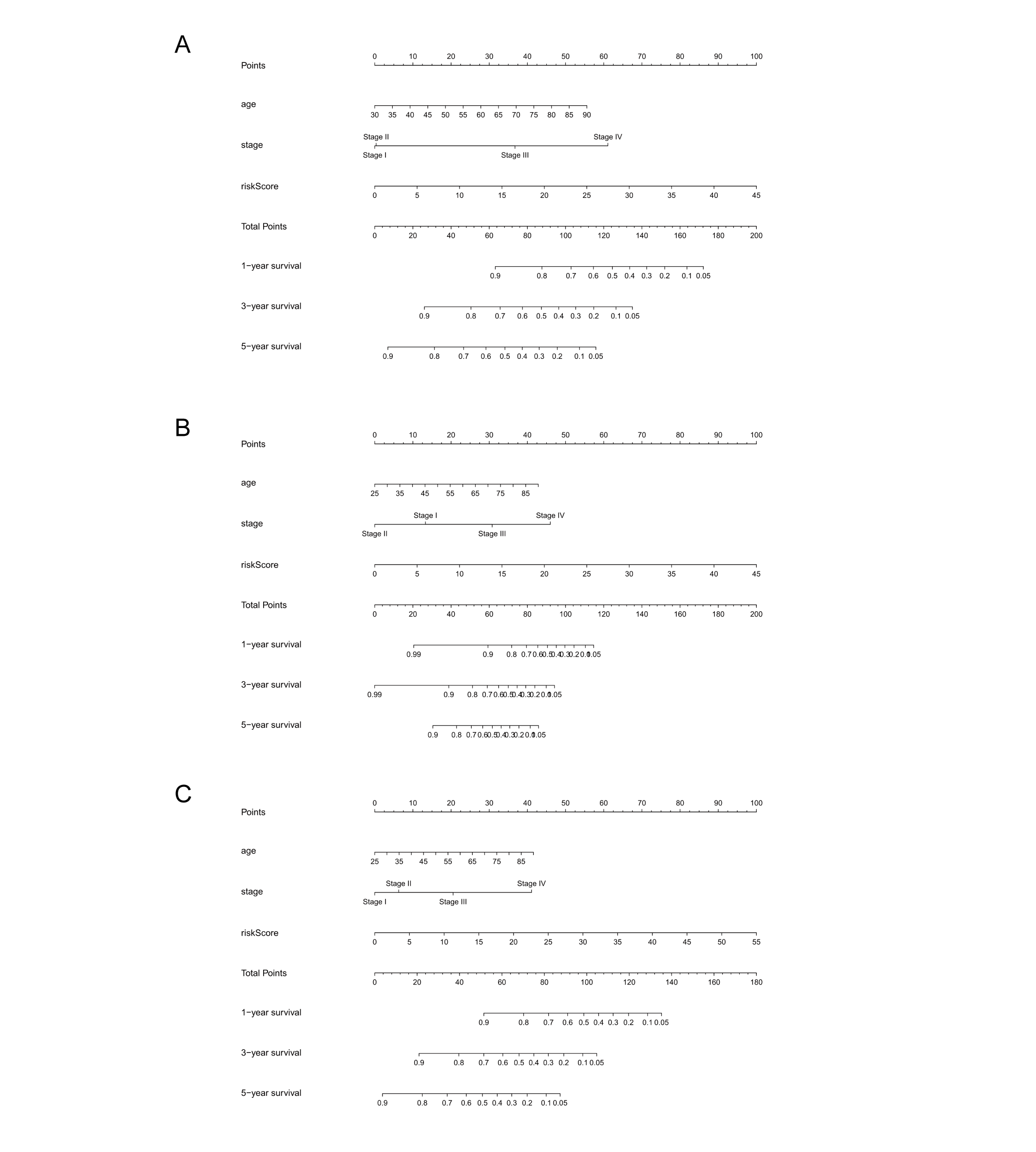

Supplement: Supplementary file 3 [file Image_3.tif]
