# Supplementary material for: An Effective Hypoxia-Related Long Non-Coding RNAs Assessment Model for Prognosis of Clear Cell Renal Carcinoma
Source: Front Oncol. 2021 Feb 22;11:616722. doi: 10.3389/fonc.2021.616722 (PMC7937891; doi:10.3389/fonc.2021.616722)
Supplement: Supplementary file 4 [file DataSheet_1.docx]

**Suplementary Table**

**TABLE 1** Relationships of the risk score and the risk genes with clinical variables in ccRCC in the 1st validation dataset

| **lncRNA symbol** | **Age**  **(≤65/>65)** | **Sex**  **(male/female)** | **Tumor grade**  **(1&2/3&4)** | **Tumor stage**  **(I & II/III & IV)** |
| --- | --- | --- | --- | --- |
| ITPR1-DT | -0.334(0.739) | -1.674(0.096) | 0.205(0.838) | -0.949(0.344) |
| AC008760.2 | 0.612(0.541) | -1.24(0.216) | -2.163(0.032) | -2.344(0.021) |
| AC084876.1 | -1.623(0.107) | 0.937(0.350) | -2.236(0.026) | -2.775(0.006) |
| AC002070.1 | 0.341(0.733) | 1.716(0.088) | 3.744(2.371e-04) | 4.717(4.262e-06) |
| LINC02027 | 1.674(0.095) | 2.917(0.004) | 0.807(0.420) | 1.076(0.283) |
| AC147651.1 | -0.093(0.926) | -0.274(0.784) | 2.312(0.022) | 0.821(0.413) |
| FOXD2-AS1 | 0.563(0.574) | -0.266(0.790) | -0.227(0.821) | -1.98(0.049) |
| LINC00944 | -1.145(0.254) | -1.597(0.112) | -3.78(1.996e-04) | -4.482(1.592e-05) |
| LINC01615 | -0.762(0.448) | -2.298(0.023) | -1.856(0.065) | -2.009(0.047) |
| Risk Score | -1.59(0.115) | -1.91(0.057) | -2.871(0.005) | -3.163(0.002) |

**TABLE 2** Relationships of the risk score and the risk genes with clinical variables in ccRCC in the 2nd validation dataset

| **lncRNA symbol** | **Age**  **(≤65/>65)** | **Sex**  **(male/female)** | **Tumor grade**  **(1&2/3&4)** | **Tumor stage**  **(I & II/III & IV)** |
| --- | --- | --- | --- | --- |
| ITPR1-DT | -0.424(0.672) | 0.954(0.344) | -1.256(0.212) | -1.218(0.228) |
| AC008760.2 | 1.384(0.168) | 0.962(0.340) | -1.657(0.100) | -1.881(0.064) |
| AC084876.1 | -0.965(0.339) | 0.158(0.875) | -1.403(0.163) | -2.061(0.042) |
| AC002070.1 | -0.444(0.659) | 0.217(0.828) | 3.966(1.181e-04) | 3.795(2.181e-04) |
| LINC02027 | 0.439(0.662) | 1.074(0.286) | 1.302(0.195) | 3.133(0.002) |
| AC147651.1 | 1.198(0.234) | 0.285(0.776) | 1.843(0.067) | 1.157(0.249) |
| FOXD2-AS1 | -0.87(0.388) | 0.325(0.746) | -0.698(0.486) | -1.976(0.051) |
| LINC00944 | -0.348(0.729) | -1.471(0.144) | -3.933(1.423e-04) | -3.985(1.553e-04) |
| LINC01615 | 0.908(0.366) | -1.65(0.102) | -1.466(0.146) | -2.039(0.046) |
| Risk Score | -0.719(0.476) | -0.137(0.891) | -3.477(7.964e-04) | -3.601(6.498e-04) |

**TABLE 3** Relationships of the risk score and the risk genes with clinical variables in ccRCC in the 3^rd^ validation dataset

| **lncRNA symbol** | **Age**  **(≤65/>65)** | **Sex**  **(male/female)** | **Tumor grade**  **(1&2/3&4)** | **Tumor stage**  **(I & II/III & IV)** |
| --- | --- | --- | --- | --- |
| ITPR1-DT | 0.905(0.366) | -2.54(0.012) | -0.821(0.412) | -1.178(0.240) |
| AC008760.2 | 1.32(0.188) | 0.004(0.997) | -3.213(0.001) | -1.809(0.072) |
| AC084876.1 | -2.083(0.039) | 0.848(0.397) | -3.29(0.001) | -3.614(3.594e-04) |
| AC002070.1 | 1.268(0.206) | 2.058(0.041) | 4.928(1.396e-06) | 4.474(1.055e-05) |
| LINC02027 | 0.12(0.905) | 3.786(2.098e-04) | 0.858(0.392) | 0.036(0.971) |
| AC147651.1 | -0.188(0.851) | 0.693(0.489) | 4.233(3.017e-05) | 1.656(0.099) |
| FOXD2-AS1 | 0.352(0.726) | 0.099(0.921) | -1.657(0.099) | -3.722(2.516e-04) |
| LINC00944 | -1.995(0.047) | -2.018(0.045) | -3.729(2.261e-04) | -4.75(3.545e-06) |
| LINC01615 | -0.425(0.671) | -2.399(0.017) | -2.436(0.016) | -2.26(0.025) |
| Risk Score | -1.373(0.171) | -0.829(0.408) | -4.144(4.672e-05) | -3.717(2.784e-04) |
